# Function and Inhibitory Mechanisms of Multidrug Efflux Pumps

**DOI:** 10.3389/fmicb.2021.737288

**Published:** 2021-12-03

**Authors:** Kunihiko Nishino, Seiji Yamasaki, Ryosuke Nakashima, Martijn Zwama, Mitsuko Hayashi-Nishino

**Affiliations:** SANKEN (The Institute of Scientific and Industrial Research), Osaka University, Osaka, Japan

**Keywords:** inhibitor, regulation, drug resistance, Gram-negative bacteria, multidrug efflux pumps

## Abstract

Multidrug efflux pumps are inner membrane transporters that export multiple antibiotics from the inside to the outside of bacterial cells, contributing to bacterial multidrug resistance (MDR). Postgenomic analysis has demonstrated that numerous multidrug efflux pumps exist in bacteria. Also, the co-crystal structural analysis of multidrug efflux pumps revealed the drug recognition and export mechanisms, and the inhibitory mechanisms of the pumps. A single multidrug efflux pump can export multiple antibiotics; hence, developing efflux pump inhibitors is crucial in overcoming infectious diseases caused by multidrug-resistant bacteria. This review article describes the role of multidrug efflux pumps in MDR, and their physiological functions and inhibitory mechanisms.

## Introduction

Multidrug resistance (MDR) is a serious problem in cancer chemotherapy and the treatment of bacterial infections. Drug resistance is often associated with drug efflux pumps decreasing cellular drug accumulation (Nikaido, 1996; Zgurskaya and Nikaido, 2000). Drug efflux pumps are membrane proteins conserved in many living organisms, including bacterial and human cells. Specifically, drug efflux pumps that recognize multiple drugs are called multidrug efflux pumps, and they cause MDR in bacteria and cancer cells. Some representative multidrug efflux pumps include AcrB in *Escherichia coli*, MexB in *Pseudomonas aeruginosa*, and *P*-glycoprotein (multidrug resistance protein 1; MDR1) in mammals. Regarding mammalian cells, research was published in 1976 on a glycoprotein that contributed to drug susceptibility in Chinese hamster ovary cells. This glycoprotein exhibited resistance to multiple drugs, and the protein found responsible was named *P*-glycoprotein (Juliano and Ling, 1976). Later, in 1986, a study describing the gene coding for *P*-glycoprotein, *mdr1*, was reported. Then in 1987, the gene was cloned from multidrug-resistant human cancer cells (Ueda et al., 1987). As for bacteria, in 1968, Nakamura determined the chromosomal region involved in resistance to acriflavine, other dyes, and SDS, and this region was named as *acrA*, to describe resistance to acriflavine (Nakamura, 1968). Later, in 1993, a gene downstream of *acrA*, called *acrB*, was cloned; AcrB was proposed as a drug efflux pump containing 12 transmembrane regions (Ma et al., 1993). Also, in 1992, the gene *emrAB*, which codes for a pump involved in MDR in *E. coli*, was also identified (Lomovskaya and Lewis, 1992).

After the genome sequence of *Haemophilus influenza* was reported in 1995, other microorganisms’ sequences were published. Genomic analysis revealed that bacteria have many genes predicted to code for drug efflux pumps (Paulsen et al., 2000). Experimental studies on these predicted genes showed that at least 20 drug efflux pump genes are present in *E. coli*, and ten in *Salmonella enterica* (Nishino and Yamaguchi, 2001a; Nishino et al., 2006b; Villagra et al., 2008). Also, X-ray crystallography and electron-microscopy imaging of multidrug efflux pumps have been used to determine the pump structures. As a result, the antibiotic recognition, transport mechanisms, and inhibitor-binding site are being better understood (Murakami et al., 2002, 2006; Nakashima et al., 2011, 2013). This review article describes the role of multidrug efflux pumps in drug resistance, their physiological function, and inhibition mechanisms, mainly for Gram-negative bacteria.

## The Role of Drug Efflux Pumps in Bacterial Drug Resistance

Mechanisms of bacterial antibiotic resistance include: (1) antibiotics inactivation via enzymatic modification or degradation, (2) changes in drug target to alter the antibiotic’s affinity, (3) changes in drug permeability through changes affecting the bacterial cell surface (e.g., by changes in the expression of outer membrane proteins), and (4) active efflux of drugs from the bacterial cells. In many cases, bacterial MDR is caused by a combination of multiple mechanisms; however, active efflux alone can achieve MDR. Multidrug efflux pumps can recognize various antibiotics with different classes of action. Due to this wide range of substrate recognition, many drugs with different molecular structures that flow into bacteria through the periplasmic space or cell membrane are prevented from reaching at their target locations, as they are actively exported from the bacterial cell by the pumps. Multidrug efflux pumps contribute to both natural and acquired drug resistance in bacteria. Analyses of clinically isolated multidrug-resistant strains show that many resistant bacteria have increased gene expression that code for multidrug efflux pumps. Thus, multidrug efflux pumps are an attractive target to be studied, with the goal of creating novel drugs [both antibiotics and efflux pump inhibitors (EPIs)] that can overcome the global threat of bacterial MDR (Blair et al., 2014).

## Families of Drug Efflux Pumps and Their Substrates

Drug efflux pumps can be classified into six different families based on the differences in their structures and coupling energies (Putman et al., 2000; Nishino et al., 2009b; Hassan et al., 2015; Kornelsen and Kumar, 2021). They include ABC (ATP-binding cassette), MF (major facilitator), RND (resistance-nodulation-division), MATE (multidrug and toxic compound extrusion), SMR (small multidrug resistance), and the relatively new family, PACE (proteobacterial antimicrobial compound efflux) (Hassan et al., 2015). Each family has a characteristic, conserved amino acid sequence. The ABC family of transporters uses the free energy released by ATP hydrolysis to ADP to facilitate the transport of its substrates across a lipid membrane in or out of the cell (Davidson and Chen, 2004). MF is the largest and most diverse superfamily of secondary transporters known to date (Law et al., 2008). It shows a variable number of transmembrane segments, with some members having 12 and others 14 transmembrane regions (Law et al., 2008). RND efflux complexes are composed of an outer membrane protein (OMP), an inner membrane protein (RND), and a periplasmic adapter protein (PAP, also known as the membrane fusion protein or MFP) that connects the OMP to the RND. The RND protein of the complex is responsible for the efflux using proton motive force (Putman et al., 2000). NorM from *Vibrio parahaemolyticus* was initially thought to be a part of the MFS family. However, due to the lack of sequence homology to any member within the MFS family, these two proteins became the first members of the MATE family of efflux pumps (Brown et al., 1999). Bacterial MATE transporters have been found to efflux cationic drugs in exchange for H^+^ or Na^+^ molecules (Omote et al., 2006). SMR family of efflux pumps is composed of small proteins with four transmembrane α-helical domains (Paulsen et al., 1996). Members of the PACE family are commonly found encoded within the core genome of a species, suggesting that these efflux pumps are perhaps involved in more than the efflux of biocides (Hassan et al., 2015).

**FIGURE 1:**
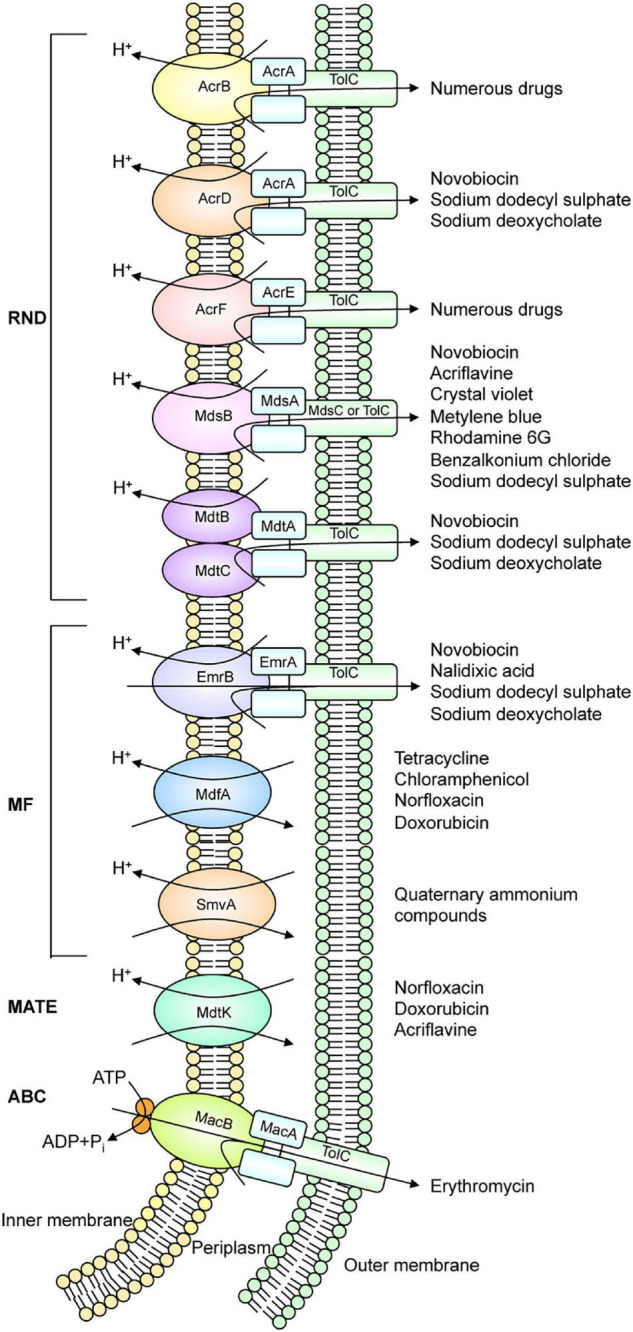
Drug efflux pump systems that exist in *Salmonella. Salmonella* has at least ten drug efflux systems.

**FIGURE 2:**
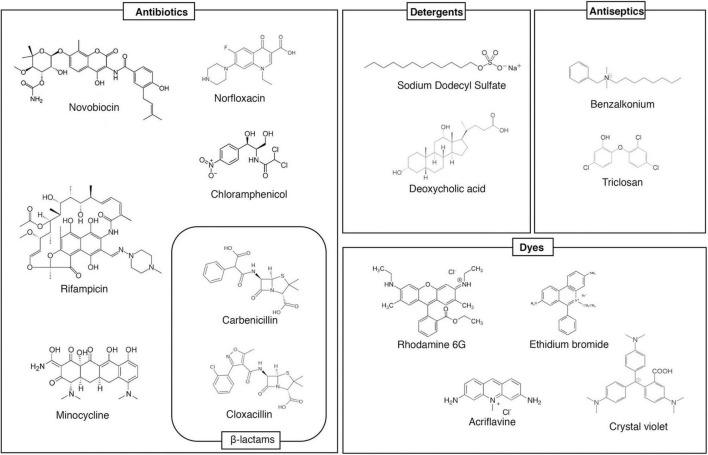
Examples multidrug efflux pump substrates. This figure shows example substrates of AcrB, a multidrug efflux pump. It recognizes many structurally unrelated antibiotics/toxins, and extrudes them from the cell, contributing to multiple drug resistance.

Homology search can estimate the number of drug efflux pump genes present in the bacterial genomes. For example, there are 37 estimated drug efflux pump genes in *E. coli*. Analysis with strains that express each efflux pump showed that at least 20 drug efflux pumps contribute to drug resistance in *E. coli* (Nishino and Yamaguchi, 2001a). Additionally, *Salmonella*, a pathogenic bacterium, has at least ten drug efflux pumps (Figure 1; Nishino et al., 2006b; Villagra et al., 2008). These efflux pumps recognize antibiotics and many other compounds with unrelated structures, such as dyes, detergents, macrolides, β-lactams, aminoglycosides, and quinolones (Figure 2; Yamasaki et al., 2016). In Gram-negative bacteria, efflux pump systems belonging to the RND family are of clinical significance. One of the most-studied multidrug efflux systems is AcrAB-TolC in *E. coli*, a tripartite complex comprising AcrB (an inner membrane RND-type protein), TolC (an OMP), and AcrA (a periplasmic protein), spanning the periplasm of bacterial cells (Zwama and Yamaguchi, 2018). Pumps that show homology with *E. coli* AcrB are MexB in *P. aeruginosa*, CmeB in *Campylobacter*, AdeB in *Acinetobacter*, and MtrD in *Neisseria gonorrhoeae*. All these pumps are involved in MDR (Blair et al., 2014). Constitutively expressed pumps contribute to intrinsic bacterial resistance to antibiotics. Also, the increased expression of pump genes contributes to acquired resistance.

## The Role of Drug Efflux Pumps in Bacterial Pathogenicity

**FIGURE 3:**
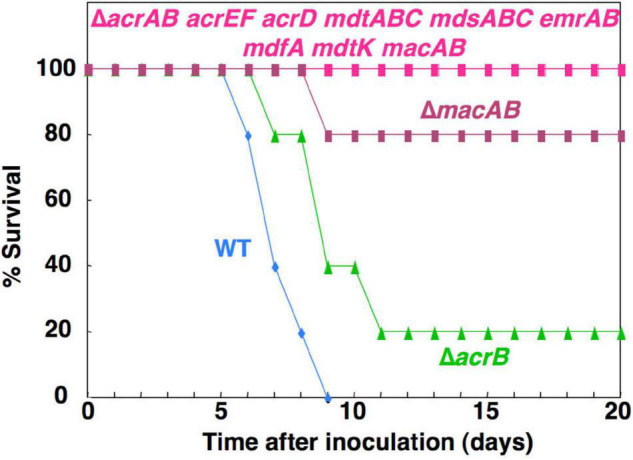
The effect of *Salmonella* drug efflux pump on the virulence. This figure shows the survival rates of BALB/c mice inoculated intragastrically with 10^6^ colony-forming units of each *Salmonella* strain.

Drug efflux pumps are often studied as factors in bacterial MDR. However, studies have shown that they are for antibiotic resistance and have physiological functions. For example, previous research demonstrated by mouse infection experiments, using *Salmonella* drug efflux pump deletion mutants, showed that drug efflux pumps contribute to the bacterial pathogenicity (Nishino et al., 2006b, 2009b; Nishino and Yamaguchi, 2008). Results showed that mice orally administered wild-type (WT) *Salmonella* died approximately within 6–9 days. However, the lethality by *Salmonella* that lacked nine drug efflux pumps was attenuated (Figure 3). The pump that contributed most to the pathogenicity was the MacAB drug efflux system, an ABC-type transporter. MacAB is thought to be an efflux system that specifically recognizes macrolide antibiotics (Kobayashi et al., 2001, 2003). However, the mice experimental results indicate that this system transports physiological substrates involved in bacterial pathogenicity or toxicity. Additionally, MacAB is regulated by PhoPQ, a two-component signal transduction system (TCS), that controls the pathogenicity of *Salmonella*; it also regulates the expression in macrophages (Zwir et al., 2005; Nishino et al., 2006b, 2009b). Furthermore, a recent study showed that linearized siderophore products secreted via MacAB efflux pump protect *Salmonella* from oxidative stress (Bogomolnaya et al., 2020).

In *E. coli*, *acr* was initially determined as the chromosomal region involved in acriflavine resistance (Nakamura, 1968). In *S. enterica*, *acrB* was identified as the gene responsible for resistance to biliary salts, detergents, and murine infection (Lacroix et al., 1996). Also, TolC of *S. enterica* is essential for the colonization of chicks (Baucheron et al., 2005), and TolC of *S. enteritidis* is required for virulence in BALB/c mice (Stone and Miller, 1995). The AcrAB-TolC efflux system of *S. enterica* plays a role in pathogenesis (Buckley et al., 2006), and the AcrB D408A mutant of this organism is attenuated in mice and *Galleria mellonella* models, showing significantly reduced invasion into intestinal epithelial cells and macrophages (Wang-Kan et al., 2017). AcrAB-TolC is also involved in the resistance, fitness, and virulence of *Enterobacter cloacae* (Perez et al., 2012).

Hirakata et al. (2002) found that the mutant of *P. aeruginosa* lacking *mexAB-oprM* is compromised in its capacity to invade or transmigrate across Madin-Darby canine kidney (MDCK) cells, and cannot kill mice. Additionally, Song et al. (2015) reported that MdsABC, *Salmonella*-specific tripartite efflux pump (Nishino et al., 2006b), showed expression-dependent alterations in the degree of resistance to extracellular oxidative stress and macrophage-mediated killing. Thin-layer chromatography and tandem mass spectrometry analyses revealed that overexpression of MdsABC led to increased secretion of 1-palmitoyl-2-stearoyl-phosphatidylserine (PSPS), affecting the ability of the bacteria to invade and survive in host cells (Song et al., 2015).

There is a large, growing body of research that demonstrates the importance of efflux pumps for bacterial colonization or infection of eukaryotic hosts (Buckley et al., 2006; Nishino and Yamaguchi, 2008; Nishino et al., 2009b; Alcalde-Rico et al., 2016; Henderson et al., 2021). Accumulating evidence that drug efflux pumps are involved in bacterial virulence indicates that these pumps are more clinically important than usually thought, and clarifying the physiological functions of these pumps is a crucial issue.

## The Role of Drug Efflux Pumps in Bacterial Iron Metabolism

Iron is essential for many biological processes, such as amino acid and nucleotide synthesis, electron transport, and peroxide reduction (Griffiths, 1991). Many bacterial species excrete iron-chelating compounds called siderophores to grow under iron-limited conditions. For example, *E. coli* can produce the catecholate siderophore enterobactin (also called enterochelin), which is a cyclic triester of 2,3-dihydroxybenzoylserine (DHBS) (O’Brien and Gibson, 1970; Pollack and Neilands, 1970). The systems responsible for enterobactin synthesis and uptake are well characterized. In contrast, the enterobactin export system is not fully understood. Additionally, drug efflux pumps, which were previously thought to be involved in drug resistance, are now known to be involved in enterobactin efflux.

**FIGURE 4:**
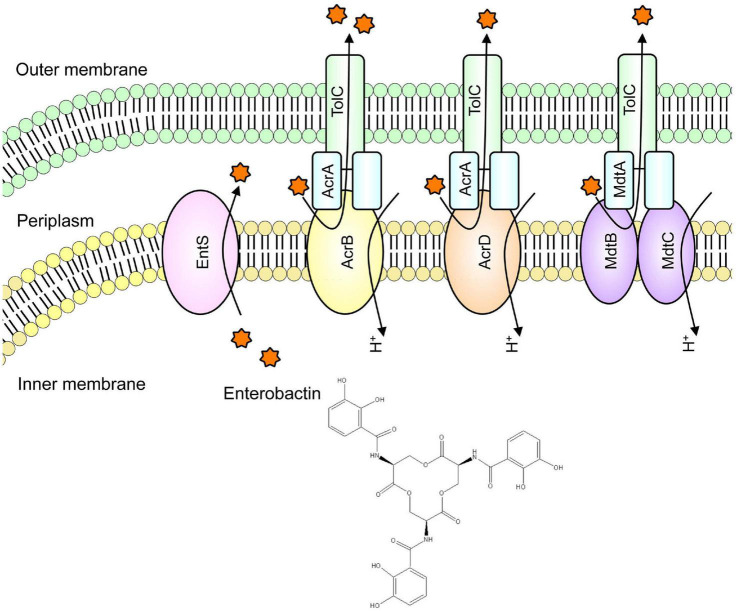
Iron chelator transport via drug efflux systems. We found that in iron-deficient conditions, enterobactin (a siderophore) is extruded out of the cell via drug efflux pumps to acquire iron for the cell.

RND-type AcrD and MdtABC are drug efflux pumps that can export antibiotics, including β-lactam, resulting in bacterial antimicrobial resistance (Nishino and Yamaguchi, 2001a; Nishino et al., 2003). In *E. coli*, it is known that siderophores are transported from the cytoplasm to the periplasm by EntS, an inner membrane protein (Furrer et al., 2002). Analysis showed that AcrD and MdtABC cooperate with AcrB to excrete a siderophore named enterobactin from the periplasm to the outside of the cell (Figure 4; Horiyama and Nishino, 2014). Iron is necessary for pathogenic bacteria in order to establish pathogenicity. As bacteria aquire iron from their hosts, they have retained systems to prevent bacterial infection by inhibiting bacterial growth by controlling the bacterial iron uptake. Therefore, siderophore secretion via drug efflux pumps is necessary for the bacteria to acquire iron from a hostile environment, indicating that this mechanism contributes to the pathogenicity.

The operon encoding the drug efflux system MexAB-OprM in *Pseudomonas aeruginosa* was initially found to be upregulated in the siderophore deficient mutant that can grow on an iron-deficient medium (Poole et al., 1993). In this report, Poole et al. showed that this operon is involved in the secretion of the siderophore pioverdin as well as in drug sensitivity of this organism (Poole et al., 1993). Furthermore, Bogomolnaya et al. (2020) recently reported that MacAB, which is related to *Salmonella* virulence, transports linearized enterobactin trimers, and its purpose may be to detoxify extracellular reactive oxygen species. Additionally, it has been reported that in *Vibrio cholerae*, the mutant lacking the RND efflux system VexGH has impaired the secretion of the catechol siderophore, vibriobactin (Kunkle et al., 2017). These results highlight the native physiological function of drug efflux pumps and provide the possibility that more drug efflux pumps may be involved in siderophore efflux and iron metabolism in multiple bacterial species.

## Roles of Multidrug Efflux Pumps in Exporting a Toxic Compound During Anaerobic Respiration

**FIGURE 5:**
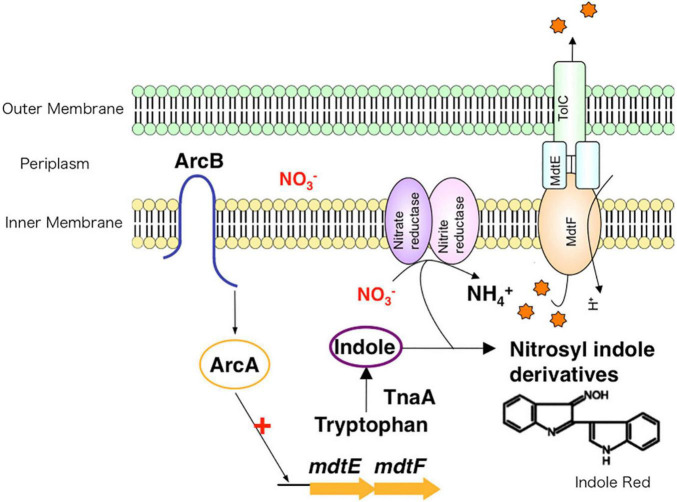
The role of drug efflux pumps in anaerobic environments. Under anaerobic conditions, nitrate respiration results in toxic compounds, such as indole red, inside of bacteria. To protect the cell from these toxins, drug efflux systems extrude toxic compounds to the outside of the cell.

The *E. coli* genome contains approximately 20 drug efflux system genes (Nishino and Yamaguchi, 2001a). However, the expression of most of these is usually suppressed under aerobic conditions, except for the tripartite system AcrAB-TolC. *E. coli* cells survive in the gut, an environment with a low oxygen concentration. However, the oxygen concentration was not previously known to affect the expression of drug efflux systems. For *E. coli*, we showed that the MdtEF drug efflux system expression is significantly induced under anaerobic conditions, and the accompanying rise in drug efflux activity leads to MDR (Zhang et al., 2011). Gene expression in *E. coli* under anaerobic and aerobic conditions is regulated by the ArcAB (anaerobic respiration control) two-component signal transduction system (TCS). The ArcA regulator recognizes a sequence upstream of the MdtEF genes. Quantitative analysis has shown that the induction of MdtEF expression under anaerobic conditions depends on ArcAB. Therefore, the expression of the MdtEF drug efflux system is regulated not by antibiotics, but by oxygen concentration changes, suggesting that this efflux system plays a physiological role in anaerobic environments. There is no difference in growth rate between the wild-type strain and an *mdtEF*-deletion strain under aerobic condition. However, under anaerobic condition, the *mdtEF*-deletion strain grew slower than the wild-type strain. Under anaerobic respiration, bacteria produce ATP using nitrate (NO_3_^–^) as the terminal electron acceptor instead of oxygen. In nitrate respiration, indole, an *E. coli* metabolite, is nitrosylated. As a result, indole derivatives such as indole red, with high toxicity, are produced during anaerobic respiration. Furthermore, we found that the expression of TnaA, an enzyme involved in indole synthesis from tryptophan, is promoted under anaerobic conditions. This causes a rise in cytosolic indole concentration compared with its production levels under aerobic growth conditions. In other words, in *E. coli* cells, anaerobic respiration results in the production of indole red, a highly toxic molecule, and consequently, the bacteria protect themselves from these toxic compounds by expressing MdtEF (Figure 5; Zhang et al., 2011). Through that research, we elucidated the mechanisms of multidrug efflux pump expression induction and their physiological functions during anaerobic respiration.

## Induction Mechanism of Multidrug Efflux Pumps by Antibiotics and Bile Acids

**FIGURE 6:**
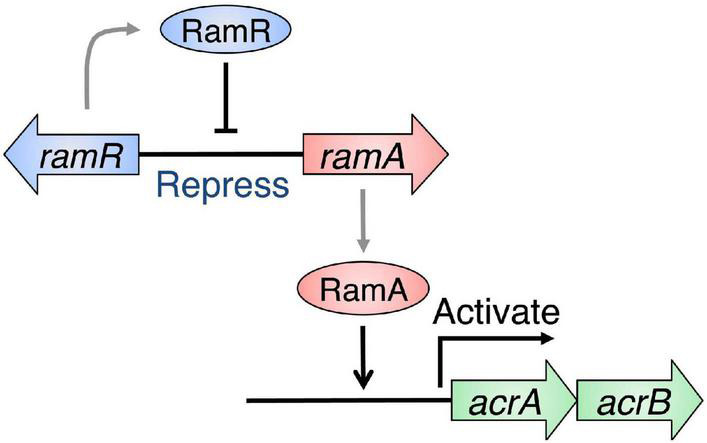
Regulation of *acrAB* multidrug efflux genes by RamRA in *Salmonella*. The expression of the multidrug efflux system genes *acrAB* is controlled by RamA, an activator protein, and RamR, a repressor. The RamR protein represses the *ramA* gene expression, which codes for the RamA protein, an expression activator in the *acrAB* drug efflux system genetic operon. The RamR protein recognizes a specific DNA sequence that exists upstream of the *ramA* gene and binds to it, repressing its expression.

**FIGURE 7:**
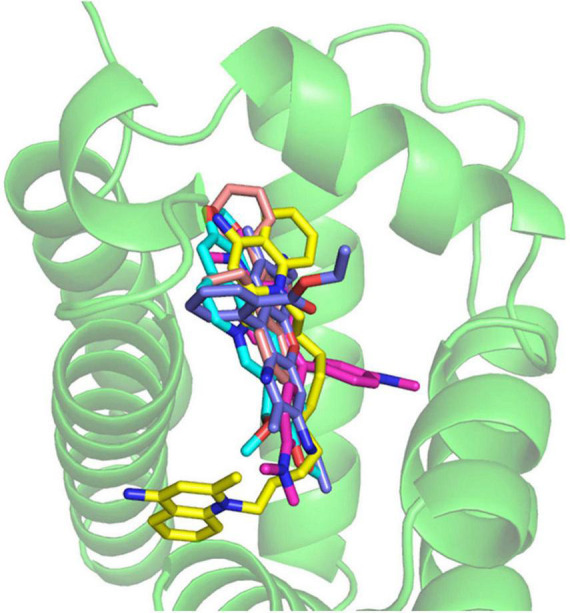
The co-crystal structure of the RamR protein with multiple cationic aromatic compounds. The RamR protein recognizes multiple compounds via multisite binding.

**FIGURE 8:**
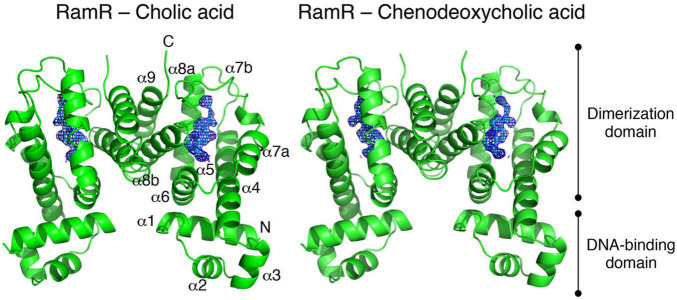
The co-crystal structure of the RamR protein with cholic acid (left) and chenodeoxycholic acid (right). The RamR protein is a dimer that binds to two cholic acid (left) or chenodeoxycholic acid (right) molecules. This binding decreases the DNA binding affinity of the RamR protein, which in turn increasing the expression of RamA, thus promoting the expression of the AcrAB drug efflux system.

Many of multidrug efflux pumps have been identified. However, the signals which induce their expression are relatively less studied. During infection, *Salmonella* cells live under various toxic environmental conditions. In the gut, where *Salmonella* infections occur, indole (produced by intestinal bacteria) and bile acids (produced by the host) act as environmental signals. Additionally, antibiotics used for infection treatments impact bacterial survival. Therefore, it is important to understand how multidrug efflux pumps are used in bacteria and how drug resistance and pathogenicity are controlled. Thus, we investigated how *Salmonella* multidrug efflux pump expression is affected by antibiotics and metabolites present in the host environment. The results showed that compounds, such as antibiotics and bile acid, reduced the DNA-binding activity of the repressor RamR, and increased the activator RamA expression. This effect contributed to the induction of the expression of the efflux pump system genes *acrAB* (Figure 6; Nikaido et al., 2008; Baucheron et al., 2012, 2014; Yamasaki et al., 2013, 2019). We solved the crystal structure of the RamR repressor, and used surface plasmon resonance (SPR) analysis to show that cholic and chenodeoxycholic acids (two of the main components of primary bile acids) bind to RamR, in addition to positively charged aromatic compounds with antimicrobial activity (such as dequalinium, berberine, crystal violet, ethidium bromide, and rhodamine 6G). We also determined the co-crystal structure of RamR with these compounds (Figures 7, 8; Yamasaki et al., 2013, 2019). We found that the various toxic compounds are recognized through multisite binding, by a combination of different amino acids in RamR. One common residue-interaction was the π–π interaction between Phe155 in RamR and the bonds in the aromatic rings in the positively charged compounds. This interaction plays a crucial role in recognizing of these compounds. However, Phe155, needed to recognize the five aromatic antibiotics, does not recognize cholic acid and chenodeoxycholic acid. Instead, hydrogen bonding with four amino acid residues (Try59, Thr85, Ser137, and Asp152) in RamR is crucial (Yamasaki et al., 2019).

**FIGURE 9:**
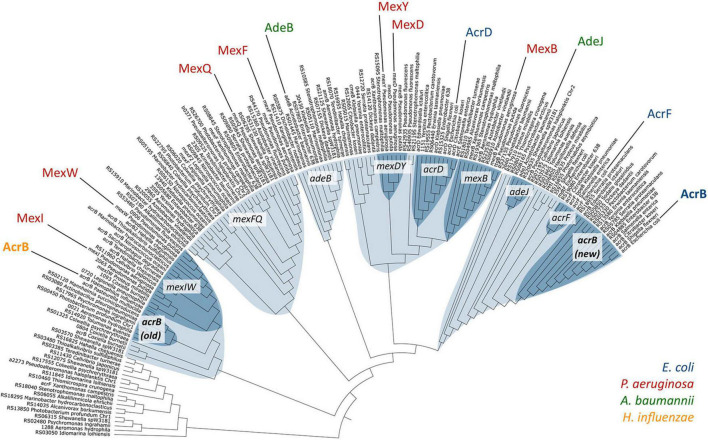
Phylogenetic relationships among a selection of RND-type MDR transporters. Different clusters are shown in light blue, and sub-clusters in dark blue. AcrB-Hi (*H. influenzae*) is on the left in yellow, and AcrB-Ec (*E. coli*) on the far right in blue (Zwama et al., 2019).

These results showed us that *Salmonella*, an intestinal bacteria, senses bile acid components via the RamR protein, and increases RamA expression, which induces the expression of the AcrAB-TolC efflux pump system. As AcrAB-TolC can expel bile acid from the cell, and the presence of bile acids controls its expression through bile-sensing regulator proteins, this mechanism is thought to be used in bile acid-rich environments, to which the cells have adapted. Also, *E. coli* expresses AcrAB-TolC to actively pump toxic compounds, including bile salts, from its environment. Furthermore, we have shown that non-intestinal bacteria, *Haemophilus influenzae*, intrinsically expresses an efflux pump (AcrAB-Hi), which could, besides exporting the same antibiotics as *E. coli* AcrAB, export bile salts only weakly (Zwama et al., 2019). Phylogenetic analysis showed that this pump is a relatively ancient efflux pump (Figure 9). These results further imply the adaptation of intestinal bacteria to bile-rich environments.

## Regulatory Networks of Multidrug Efflux Pumps

Bacteria are known to respond to different environments via a two-component signal transduction system. This two-component system consists of an environmental sensor (histidine kinase) and an intracellular control factor (response regulator) (Hoch, 2000). The sensors detect any specific environmental signals, and the specific histidine residue is self-phosphorylated. This phosphate group is then transferred to the specific aspartic acid in the response regulator. The regulator mostly acts as a transcriptional regulator to control the expression of genes that have various activities regarding biological reactions (Zwir et al., 2005; Nishino et al., 2006a). Genome analysis revealed the existence of 30 varieties of two-component signal transduction systems encoded in the chromosomes of *E. coli* (Mizuno, 1997). Hirakawa et al. (2003) cloned all of the regulators of the two-component system in *E. coli*; then, the effects of the regulators on drug sensitivities of *E. coli* were tested. Results showed that 15 of the regulators were detected to be involved in the drug resistance (Hirakawa et al., 2003). Three two-component signal transduction systems, EvgSA, BaeSR, and CpxAR, are capable of inducing efflux pump expression, resulting in increased MDR. The EvgSA system induces the expression of the MdtEF pump and increasing the level of MDR (Nishino and Yamaguchi, 2001b,^2002^). The BaeSR signal transduction system is activated through indole, copper or zinc, resulting in the induction of the two pumps, MdtABC and AcrD, making bacteria multidrug- and metal-resistant (Nagakubo et al., 2002; Nishino et al., 2005, 2007). The CpxAR system is a two-component signal transduction system that responds to membrane stress, and causes MDR. We found the overproduction of NlpE, an outer membrane lipoprotein functioning during envelope stress responses, increases multidrug and copper resistance by activating the genes encoding the AcrD and MdtABC multidrug efflux pumps (Nishino et al., 2010).

Although the expression of multidrug efflux pumps is activated by two-component signal transduction systems, they are also controlled by repressors. AcrR and AcrS repress the expression of *acrAB* (Ma et al., 1996; Hirakawa et al., 2008) in *E. coli*. EmrR is reported to be a repressor for the *emrAB* efflux genes (Lomovskaya et al., 1995). CRP also represses the *mdtEF* genes (Nishino et al., 2008). The histone-like protein H-NS is a major component of the bacterial nucleoid and plays a crucial role in the global gene regulation of enteric bacteria (Jacquet et al., 1971; Bloch et al., 2003). It was found that H-NS represses the drug efflux genes, *acrEF*, *mdtEF*, and *emrKY* of *E. coli* (Nishino and Yamaguchi, 2004), and *acrEF* of *Salmonella* (Nishino et al., 2009a). It was also found that small non-coding DsrA RNA modulates multidrug efflux through activation of genes encoding the MdtEF pump in *E. coli* (Nishino et al., 2011). It was shown that the RNA chaperone Hfq positively regulates the production of the AcrB drug efflux protein in *E. coli* (Yamada et al., 2010) and Hfq also plays a role in *Salmonella* intrinsic acriflavine resistance, and the SmvA efflux pump is involved in this resistance (Hayashi-Nishino et al., 2012).

As described above, the AcrAB-TolC efflux system is effective in generating drug resistance and has wide substrate specificity. The expression of *acrAB* is subject to multiple levels of regulation. It is modulated locally by the repressor AcrR (Ma et al., 1996). At a more global level, it is modulated by stress conditions and by regulators such as MarA, SoxS, and Rob (Alekshun and Levy, 1997; White et al., 1997; Martin and Rosner, 2002, 2003; Randall and Woodward, 2002; Rosenberg et al., 2003). A study demonstrated that AcrAB is also positively regulated by SdiA, a protein that regulates cell division genes in a manner dependent upon quorum sensing (Rahmati et al., 2002). Yang et al. (2006) suggested that drugs exported by pumps may resemble communication molecules normally exuded.

## Structure of Drug Efflux Pump AcrB and Antibiotic Transport Mechanism

**FIGURE 10:**
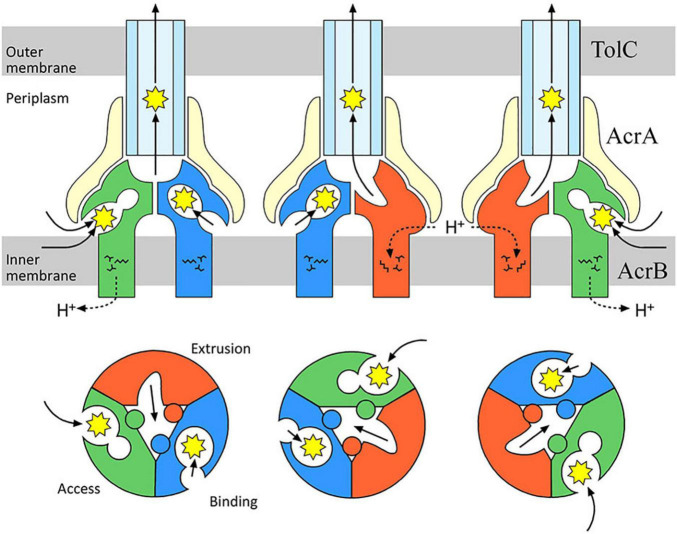
Drug transport via the AcrAB-TolC multidrug efflux system. AcrB facilitates the proton motive force as its energy source to extrude drugs from the periplasm or inner membrane to the outside of the cell. During drug-transport, each monomer of the AcrB trimer has a different structure. Drugs are transported in sequence from the Access to the Binding monomer, and then to the Extrusion monomer. We found a drug-recognition pocket near the entrance called the Proximal Binding Pocket (PBP, expanded in the Access monomer), and a Distal Binding Pocket (DBP) near the exit (expanded in the Binding monomer). Erythromycin (molecular weight: 734 g mol^–1^) and rifampicin (molecular weight: 823 g mol^–1^), two drugs with a relatively large molecular weights, temporarily bind to the PBP, then they are sent to the DBP via a peristaltic pump mechanism (Nakashima et al., 2011).

After the crystal structure of AcrAB-TolC component TolC from *E. coli* was obtained (Koronakis et al., 2000), Murakami et al. (2002) published the first crystal structure of and an RND-type transporter AcrB. This structure of AcrB was a symmetrical homotrimer with identical monomer conformations. However, in 2006, the co-crystal structure analysis with small molecular mass drugs, such as minocycline (molecular weight: 457 g mol^–1^) and doxorubicin (molecular weight: 544 g mol^–1^), showed these drugs bound to only one of the monomers in the homotrimer (this monomer was called the Binding monomer). These results showed that the three AcrB monomers had different conformations (Murakami et al., 2006). According to the structure, the three monomers were named the Access, Binding, and Extrusion monomer, and the cycling between these three states to transport drugs was proposed as the “functionally rotating mechanism” (Figure 10). Nakashima et al. (2011) reported the results of their co-crystal structure analysis of AcrB bound with large molecular mass drugs erythromycin (molecular weight: 734 g mol^–1^) and rifampicin (molecular weight: 823 g mol^–1^). Here, the structure of AcrB itself was virtually identical to that reported in 2006. However, there was major difference in the binding locations of the drugs. Minocycline and doxorubicin bound in a phenylalanine-rich pit in the Binding monomer, but rifampicin and erythromycin were bound at a location closer to the entrance of the pump in the Access monomer. These two distinct binding pockets were named the Distal Binding Pocket (DBP) and the Proximal Binding Pocket (PBP), respectively. They were separated by a short loop with Phe617 at its tip that existed between the pockets. We found that this loop swings widely during the transition from the Access to the Binding conformational states. Drugs, such as erythromycin, that bind to the PBP are thought to be transported to the DBP by a series of structural changes, including the swinging of the previously mentioned loop. Additionally, a highly flexible hinge, called the Hoising-loop, changes its conformation significantly during the transition to and from the Extrusion state (from a random coil in the Binding monomer, to an α-helix in the Extrusion monomer, and back to a random coil in the Access monomer) (Murakami et al., 2006; Zwama et al., 2017). High molecular mass drugs temporarily bind to the Access monomer and later are transported to the DBP (during the transition to the Binding state). Finally, they are extruded from the exit by the extrusion monomer. We clarified the stepwise mechanism by which drugs are transported from the PBP to the DBP, and then to the exit. This extrusion of antibiotics from the cell has also been described as a peristaltic motion (Seeger et al., 2006; Figure 10). Besides the existence of multiple pockets and multisite binding to multiple antibiotics and toxic compounds, we have shown evidence for the existence of multiple entrance channels within the monomer of the AcrB efflux pump which contribute to the recognition of a wide range of structurally different compounds (Murakami et al., 2002, 2006; Nakashima et al., 2011; Zwama et al., 2018).

## Drug Efflux Pump Inhibition Mechanism

**FIGURE 11:**
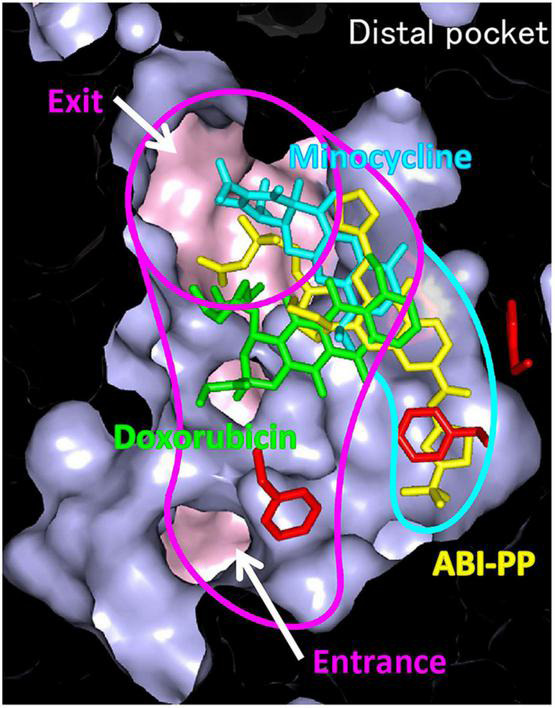
Differences in the binding patterns of drug efflux pump to inhibitor, ABI-PP. Efflux pump inhibitor ABI-PP, penetrated and bound to a narrow hydrophobic domain that branches off from the substrate transport pathway, confirming the existence of an inhibitor-binding pit (Nakashima et al., 2013).

The inhibition of efflux pumps appears to be a promising strategy to restore antibacterial potency because active efflux of antibacterial agents plays a significant role in mediating drug resistance in bacteria (Wang et al., 2016). One existing EPI is the pyridopyrimidine derivative ABI-PP. ABI-PP inhibits the function of MexB from *P. aeruginosa*, but cannot inhibit MexY (Nakashima et al., 2013). MexY is another clinically relevant RND-type efflux pump known to be expressed in multidrug-resistant *P. aeruginosa*. Partly because of this, ABI-PP is not clinically usable. Co-crystal structure analysis of ABI-PP with *E. coli* AcrB and *P. aeruginosa* MexB showed that, similar to the pump substrates minocycline and doxorubicin, ABI-PP is bound to the DBP in the Binding monomer. However, unlike the substrate drugs, ABI-PP is bound tightly into a narrow hydrophobic region that branches off from the pocket, indicating the existence of an inhibitor-binding pit (Figure 11; Nakashima et al., 2013). This phenomenon indicates that ABI-PP suppresses the functional rotational peristaltic motion of the pump by strongly binding to the hydrophobic pit that branches off from the DBP. One phenylalanine residue halfway into the AcrB and MexB inhibitor-binding pit (Phe178) is replaced by tryptophan in MexY (Trp177); an amino acid with a bulky side-chain. Homology modeling analysis showed that this large side-chain causes steric hindrance, implicating the reason ABI-PP is unable to bind to MexY. Using mutant variants suggests that minute steric hindrances determine the specific inhibition of ABI-PP in the binding pit (Nakashima et al., 2013). These results help to the design of EPIs that will avoid steric hindrance, and develop universal EPIs that can inhibit various multidrug efflux pumps.

## Conclusion

Bacterial drug efflux pumps are ideal targets for developing inhibitors (EPIs), and the research described in this review may help overcome MDR and help create reusable antibiotics. Furthermore, there are more studies on how multidrug efflux pumps contribute to physiological pathogenicity and biofilm formation. Hence, inhibitors may hold promise in reducing these phenomena as well. PAβN and ABI-PP are two representative inhibitors that have been reported. PAβN is not used clinically due to its toxicity to eukaryotic cells. Also, ABI-PP binds to the DBP and the hydrophobic domain that branches off from the DBP in AcrB in *E. coli* and MexB in *P. aeruginosa*. This binding of ABI-PP inhibits the functional rotation of some efflux pump, but cannot inhibit MexY-like pumps, which are highly expressed in multidrug-resistant *P. aeruginosa*. Thus, this EPI is not clinically effective, either (Nakashima et al., 2013). Multiple RND-type efflux pumps exist inside Gram-negative bacteria. Therefore, it is thought that the development of a compound that can simultaneously inhibit the function of multiple major pumps (a universal inhibitor) is necessary for overcoming MDR. Understanding efflux pump structures and the pump mechanism (including the substrate recognition and drug-extrusion mechanisms) can help us to design more effective EPIs. In addition to PAβN and ABI-PP, there are many reports on EPIs from pharmaceutical companies and research groups (Wang et al., 2016). However, they have not yet been used clinically. Inhibiting drug efflux pumps allows existing antimicrobial agents to accumulate more efficiently in bacterial cells, which is an effective approach to overcome bacterial resistance. However, the pharmacokinetics of the two compounds must be matched to use existing antimicrobial agents with pump inhibitors in treating infectious diseases. Therefore, it will be necessary to modify the compounds to match their pharmacokinetics. Additionally, because there are multiple pumps in a single bacterial cell, it is challenging to combat resistant bacteria by inhibiting only one pump, considering when multiple pumps are expressed simultaneously. Discovering the common point of multiple pumps for their function and developing inhibitors that target this point is necessary to overcome these problems. For example, in the case of the RND efflux system, since it functions by forming a complex, compounds that inhibit the complex formation might effectively inhibit the pump. In addition to the screening of small molecule compounds, it would be worthwhile to try methods, such as screening of peptide inhibitors or methods genetically preventing the expression of efflux pumps. In any case, if it becomes possible to inhibit the pump, bacteria become susceptible to various drugs, and developing more efficient inhibition methods will help overcome drug resistant bacterial infections.

## Author Contributions

KN, MZ, and MH-N wrote the manuscript. KN, MZ, RN, and SY prepared the figures. KN edited the manuscript. All the authors have read and agreed to the published version of the manuscript.

## Conflict of Interest

The authors declare that the research was conducted in the absence of any commercial or financial relationships that could be construed as a potential conflict of interest.

## Publisher’s Note

All claims expressed in this article are solely those of the authors and do not necessarily represent those of their affiliated organizations, or those of the publisher, the editors and the reviewers. Any product that may be evaluated in this article, or claim that may be made by its manufacturer, is not guaranteed or endorsed by the publisher.
